# Synchronization of Biological Clock Neurons by Light and Peripheral Feedback Systems Promotes Circadian Rhythms and Health

**DOI:** 10.3389/fneur.2015.00128

**Published:** 2015-06-05

**Authors:** Ashna Ramkisoensing, Johanna H. Meijer

**Affiliations:** ^1^Laboratory for Neurophysiology, Department of Molecular Cell Biology, Leiden University Medical Center, Leiden, Netherlands

**Keywords:** SCN, electrophysiology, diurnal, disease, aging, exercise, photoperiod, plasticity

## Abstract

In mammals, the suprachiasmatic nucleus (SCN) functions as a circadian clock that drives 24-h rhythms in both physiology and behavior. The SCN is a multicellular oscillator in which individual neurons function as cell-autonomous oscillators. The production of a coherent output rhythm is dependent upon mutual synchronization among single cells and requires both synaptic communication and gap junctions. Changes in phase-synchronization between individual cells have consequences on the amplitude of the SCN’s electrical activity rhythm, and these changes play a major role in the ability to adapt to seasonal changes. Both aging and sleep deprivation negatively affect the circadian amplitude of the SCN, whereas behavioral activity (i.e., exercise) has a positive effect on amplitude. Given that the amplitude of the SCN’s electrical activity rhythm is essential for achieving robust rhythmicity in physiology and behavior, the mechanisms that underlie neuronal synchronization warrant further study. A growing body of evidence suggests that the functional integrity of the SCN contributes to health, well-being, cognitive performance, and alertness; in contrast, deterioration of the 24-h rhythm is a risk factor for neurodegenerative disease, cancer, depression, and sleep disorders.

## Introduction

The rotation of the Earth around its central axis causes a daily rhythm in environmental factors, including light intensity, temperature, and food availability. In order to anticipate these 24-h changes in the environment, many species have evolved an internal clock. In mammals, this internal clock resides in the suprachiasmatic nucleus (SCN) in the ventral hypothalamus (1). The SCN is a bilateral structure containing 20,000 neurons that generate circadian rhythms. The SCN synchronizes its circadian rhythm to the external day–night cycle using light information that is projected via the retinohypothalamic tract (RHT). This information is then conveyed to other regions in the central nervous system (2, 3). Based on neuropeptide expression, the SCN is sub-divided into the dorsal (i.e., shell) and ventral (i.e., core) SCN (4–7). The ventral SCN expresses gastrin-releasing peptide (GRP) and vasoactive intestinal polypeptide (VIP) (1, 8), whereas the dorsal SCN contains the hormone vasopressin (9). Moreover, the dorsal SCN receives strong input from the ventral SCN (9), whereas the ventral SCN receives little input from the dorsal SCN (10).

Individual cells in the SCN generate a circadian rhythm via a series of interconnected positive and negative feedback loops; these feedback loops regulate the transcription and activity of clock genes and proteins, respectively (11, 12). One feedback loop is regulated by the transcription factors Circadian Locomotor Output Cycles Kaput (CLOCK) and Brain and Muscle ARNT-like protein 1 (Bmal1). These proteins drive the transcription of specific target genes in the Period (*Per1*, *Per2*, and *Per3*) and Cryptochrome (*Cry1* and *Cry2*) gene families; in turn, Per and Cry proteins inhibit CLOCK/Bmal1-mediated transcription. Another feedback loop consists of the nuclear receptors ROR (α, β, and γ), PPARα, and REV-ERB (α and β). Dissociated SCN cells retain a circadian rhythm in their electrical firing rate, with a relatively wide range of intrinsic periods (ranging from 22 to 28 h) (13–15). This ability of isolated neurons to maintain their intrinsic rhythm indicates that individual SCN neurons function as autonomous single-cell oscillators driven by intrinsic molecular feedback loops (14). The key implication of this finding is that the SCN’s multicellular structure depends upon cooperation among individual neurons in order to function effectively as a coherent pacemaker. In this review, we will discuss the mechanisms that underlie synchronization, and we will discuss the relevance and significance of synchronization for health and disease. Next, we will discuss the consequences of disrupted SCN synchronization on aging, sleep disorders, neurodegenerative diseases, and metabolic disorders. In addition, positive effects of physical exercise on SCN rhythm amplitude will be discussed. Finally, we will compare the organization of the SCN of nocturnal and diurnal species with special focus on potential differences in synchronization mechanisms.

## Synchronization of SCN Neurons

### Phase shifts in the SCN are driven by synchronized SCN neurons

Both *in vitro* and *in vivo* recordings of SCN firing frequency revealed that the SCN’s electrical activity output has a sinusoidal-like waveform pattern that peaks during the subjective day and is low during the subjective night (16, 17). In nocturnal animals, the trough of the SCN’s electrical activity corresponds with the animal’s behaviorally active phase (18–20). In diurnal animals, this relationship between electrical activity and behavioral activity is reversed; thus, the SCN’s activity peaks in phase with the behaviorally active phase (21). Recordings in the SCN of freely moving mice revealed close correspondence between the SCN’s pattern of electrical activity and the animal’s behavioral activity. Specifically, the behavioral transitions from rest to activity – and vice versa – occur at the mid-point in the declining and increasing slopes in SCN activity, respectively (22). The most intense level of behavioral activity occurs during the trough in electrical activity rhythm, and silencing activity in the SCN by applying tetrodotoxin during the animal’s resting phase induces behavioral activity (23).

A subpopulation of SCN neurons (comprising 32 and 38% of SCN neurons in rats and hamsters, respectively) exhibit light-induced changes in electrical activity (24–29). At low intensities (0.1 lux in rats and 1 lux in hamsters), light can suppress electrical activity, whereas high light intensity increases the rate of neuronal firing in an intensity-dependent manner (25). In response to external light, the clock genes *Per1* and *Per2* are induced in the SCN (30–34), and the duration of this induction is dependent upon the intensity of the light (35). Glutamate is the primary neurotransmitter used by the RHT to project light information to the SCN (36). The application of glutamate – or agonists of the glutamate receptor – to the SCN causes a phase shift in the SCN’s electrical activity that mimics light-induced phase shifts in behavior (37–39) and alters the levels of *Per1* and *Per2* mRNA (34). The SCN’s rhythm is synchronized to the daily light–dark cycle by the phase-shifting effects of light on the SCN. Early in the subjective night, light has a phase-delaying effect; in contrast, light in the late subjective night causes a phase advance (40–42). These phase-dependent responses are fundamental to the animal’s ability to entrain to a new light cycle, and are present in all living organisms that exhibit circadian rhythmicity. Whether a light-induced phase delay or phase advance will occur depends upon intercellular signaling cascades and is therefore an intrinsic property of the SCN (43–46).

Following exposure to a shift in the light–dark cycle (for example, by crossing time zones), the SCN generally takes several cycles to resynchronize (47–52). Recordings of the SCN’s firing rate in the rat revealed that delay-shifting the light–dark cycle by 6 h induces a transient bimodal electrical activity rhythm in the SCN (47). One component of this bimodal activity pattern reflects the activity of a group of neurons that synchronize immediately to the new light–dark cycle, whereas the other component reflects the activity of neurons that remain synchronized to the previous light–dark cycle. Separation of the ventral SCN from the dorsal SCN by surgical incision revealed a unimodal electrical activity pattern in both regions and revealed that the shifted and non-shifted components are generated by the ventral and dorsal SCN, respectively. Surprisingly, following a shift in the light–dark cycle, the electrical activity profile of the ventral SCN (i.e., the shifted component) is considerably more narrow than the electrical activity profile of the dorsal SCN (i.e., the non-shifted component). Curve-fitting analysis revealed that the narrow, shifted component is composed of the electrical output produced by only 20% of the entire SCN’s neuronal population (53). Furthermore, simulations revealed that the ventral SCN’s narrow electrical activity profile is not due to the low number of neurons that contribute to this component, but is actually the result of high synchrony among these neurons (54). The differences in light-induced phase shifts between the ventral and dorsal SCN could be the result of differential innervation by the optic nerve (5, 55, 56). In rats, the ventral SCN receives the majority of light input projected by the retina (3, 6, 7) and has more pronounced light-evoked changes in terms of electrical activity (57, 58) and gene expression (59–64). In contrast, the dorsal SCN is only sparsely innervated by the retina (1).

### Robustness of the SCN’s output is correlated with synchrony among SCN neurons

**Figure 1:**
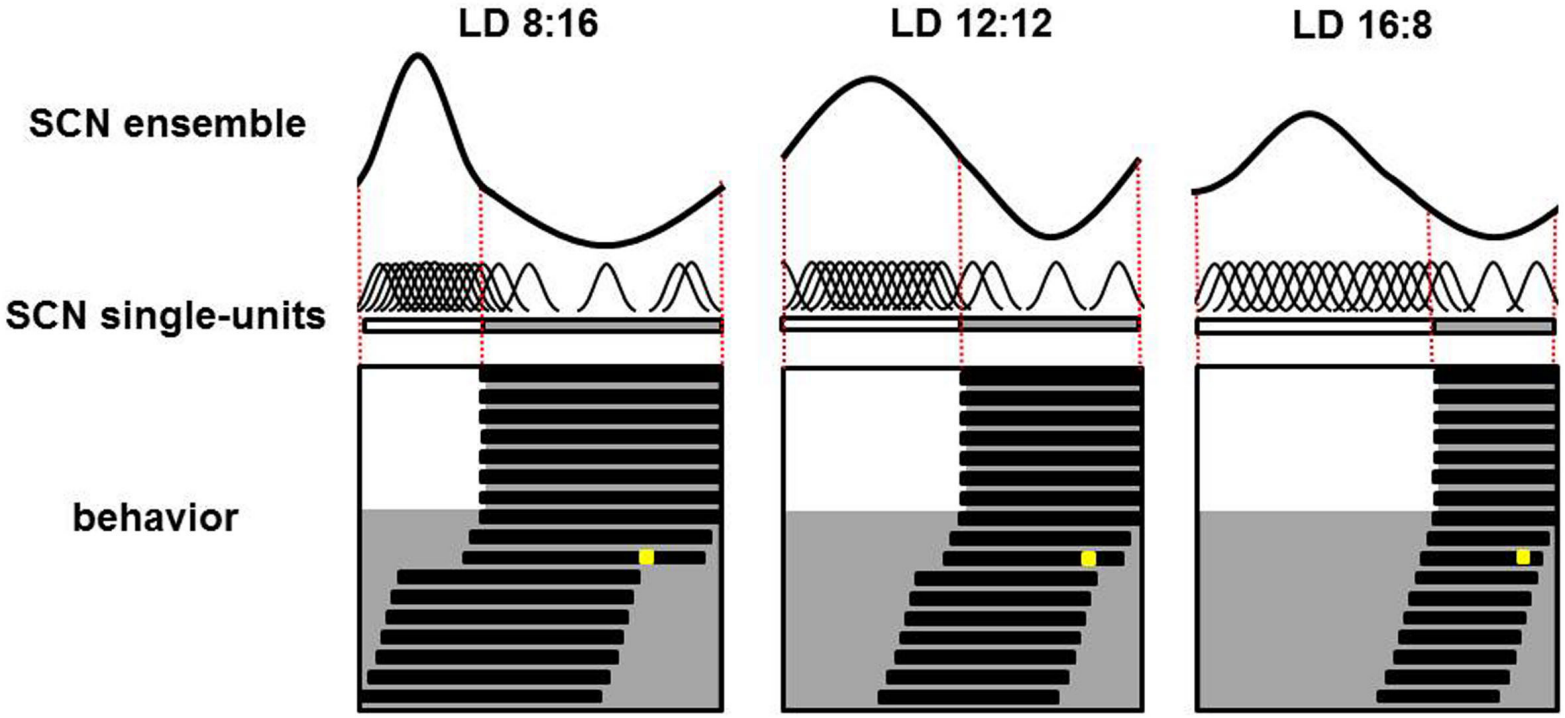
**Plasticity of the SCN**. Schematic overview of the SCN’s ensemble rhythm (top), single-cell activity in individual SCN neurons (middle), and the animal’s behavioral activity pattern (bottom) in a nocturnal rodent entrained to a short (LD 8:16; left column), medium (LD 12:12; middle column), or long (LD 16:8; right column) photoperiod. In the lower panels, days are depicted on successive lines, and light and dark periods are represented by white and gray backgrounds, respectively. Following a transition to continuous darkness, aftereffects of the photoperiod on behavioral activity can be detected. Following a short photoperiod, the animal’s active phase is longer; in contrast, following a long photoperiod, the active phase is shorter. A light pulse applied late in the subjective night in the third cycle of continuous darkness (the yellow square) results in a large-magnitude phase advance in animals entrained to a short photoperiod, a smaller phase advance in animals entrained to a medium photoperiod, and virtually no phase advance in animals entrained to a long photoperiod (phase advance is measured as the difference in activity onset before and following the light pulse). For each photoperiod, the SCN’s ensemble electrical activity rhythm is depicted in the top row. The relative amplitude of the SCN ensemble rhythm is large in short photoperiods and small in long photoperiods; this difference is due to different levels of synchronization among the electrical activity patterns of the individual neurons. The single-unit activity traces are highly synchronized in animals entrained to a short photoperiod, and less synchronized in animals entrained to a long photoperiod (middle row).

*In vivo* recordings of SCN electrical activity revealed that the SCN’s waveform pattern differs between long and short photoperiods (65). The changes in the SCN’s electrical activity waveform in response to a change in photoperiod correspond with observable behavioral adaptations. In both long and short photoperiods, the onset and offset of behavioral activity occur at the mid-point threshold in the declining and rising slopes of electrical activity, respectively (65). Even in continuous darkness, the SCN retains its intrinsic photoperiod-induced waveform for several cycles, suggesting that the SCN has a photoperiodic memory. Furthermore, the photoperiod-induced waveform is preserved even after the SCN has been isolated *in vitro*. After entraining to a short photoperiod, the SCN’s ensemble discharge rate has a waveform with a short duration of enhanced activity; in contrast, after entraining to a long photoperiod, the SCN’s waveform has a long period of enhanced activity (65, 66). Computational studies showed that changes in phase-synchronization are extremely effective in terms of inducing a change in waveform, whereas changes in single-cell activity patterns have relative weak effects (67, 68). Experimental studies have confirmed that nature indeed functions in this way. Both single-cell and subpopulation recordings revealed that after entraining to a short photoperiod, the activity of individual SCN neurons is more synchronized and clusters around subjective midday. On the other hand, after entraining to a long photoperiod, the activity of SCN neurons is less synchronized (Figure 1).

In the dorsal SCN, the electrical activity patterns differ somewhat between short and long photoperiods; however, computational studies revealed that these differences in single-cell activity patterns in the dorsal SCN are not sufficient to explain the waveform changes that occur at the network level (67, 69). However, the narrow phase distribution of subpopulation activity during short photoperiods does explain the narrow peak width in the SCN’s waveform, whereas the broad phase distribution during long photoperiods explains the broad peak width in the SCN’s waveform (65, 66). These findings are supported by molecular studies that show similar single-cell *Per1* expression patterns after entrainment to either long or short photoperiods (70). The results from electrophysiological, computational, and molecular studies indicate that synchrony between individual SCN neurons – rather than a change in the activity pattern of those individual neurons – is important for adapting to a change in photoperiod.

### Amplitude of the SCN’s output correlates with the SCN’s phase-shifting response

In the 1980s, Pittendrigh and colleagues reported that the photoperiod to which hamsters were entrained affected the resulting amplitude of the phase-response curve. Thus, hamsters that were entrained to a short photoperiod had a larger light-induced phase shift compared to hamsters that were entrained to a long photoperiod (71). More recent studies support these early findings (72–74). The difference in the light-induced behavioral phase shift between short and long photoperiods is not the result of a difference in retinal response; rather, the difference occurs at the level of the SCN. Bath application of the glutamate receptor agonist *N*-methyl-d-aspartate (NMDA) to an SCN entrained to a short photoperiod causes a significantly larger phase delay compared to an SCN that was entrained to a long photoperiod (74). Moreover, the acute effect of applying a pulse of NMDA is similar between a short photoperiod-entrained SCN and a long photoperiod-entrained SCN, suggesting that the difference in the phase delay in the SCN is not caused by desensitization to glutamate in long photoperiods (74); thus, another mechanism must explain these results.

The amplitude of the SCN’s electrical rhythm is high when the neurons in the SCN are more synchronized (i.e., when entrained to a short photoperiod), whereas the amplitude of the rhythm is low when the neurons are less synchronized (54, 65, 67, 75). Results from both behavioral and *in vitro* studies revealed that an SCN with high amplitude exhibits a larger shift in response to a given perturbation compared to an SCN with a low rhythmic amplitude (i.e., from a long photoperiod) (74). These results are surprising, as they do not intuitively match predictions that arise from limit cycle oscillator theory, a theory that is often used to model the phase-shifting behavior of oscillators. The limit cycle model predicts that following a perturbation of a given magnitude, oscillators that oscillate with a high amplitude will shift to a lesser degree than oscillators that oscillate with a lower amplitude (71, 76, 77). This prediction holds true for primitive organisms such as *Gonyaulax* (78) and *Neurospora* (79, 80). However, the experimental finding that an SCN with a high-amplitude oscillation shifts to a greater extent than an SCN with a low-amplitude oscillation is not consistent with the predictions of limit cycle theory. This apparent discrepancy between theory and practice may be due to the SCN’s functioning at the level of a network. In a population of highly synchronized neurons, each individual neuron will be more in phase, and an external perturbation of the system will cause a similar phase-shifting response in the individual cells, thereby driving a large net shift in the SCN network. In a relatively desynchronized SCN, the individual neurons will be out of phase, and an external perturbation will induce different phase-shifting responses among the individual neurons, thereby causing a relatively small net shift in the SCN network (81). Simulations have confirmed this prediction with surprisingly high accuracy (62). In Afh/Afh mice, the amplitude of the SCN ensemble is reduced by a reduction of the amplitude of single-cell oscillations (82). In accordance to the limit cycle theory, the Afh/Afh mice show high-amplitude resetting to light. To explain light-resetting by the SCN, both the amplitude of single-cell oscillations as well as phase-synchronization among single cells should be taken into consideration.

Under certain conditions, the SCN – as a network – can behave as a limit cycle oscillator. Simulation studies showed that the phase-shifting response of the SCN is opposite to the predictions of a limit cycle oscillator if just a fraction of the network is directly influenced by the perturbation (e.g., if light affects only 20% of the population). On the other hand, if all of the neurons in the SCN network are affected by the perturbation (for example, a change in temperature, which would affect 100% of the neurons in the population), the SCN can exhibit the behavior predicted by limit cycle oscillator theory (83). Thus, although limit cycle theory accurately predicts the behavior of an *individual* oscillator, the phase-shifting behavior of an entire *network* of oscillators is more difficult to predict, as such behavior is dependent upon the degree of synchrony among the individual oscillators and the percentage of neurons that will respond to the perturbation (i.e., a 100% response rate to temperature vs. a 20% response rate to external light).

### Role of chemical coupling in SCN neuronal synchronization

**Figure 2:**
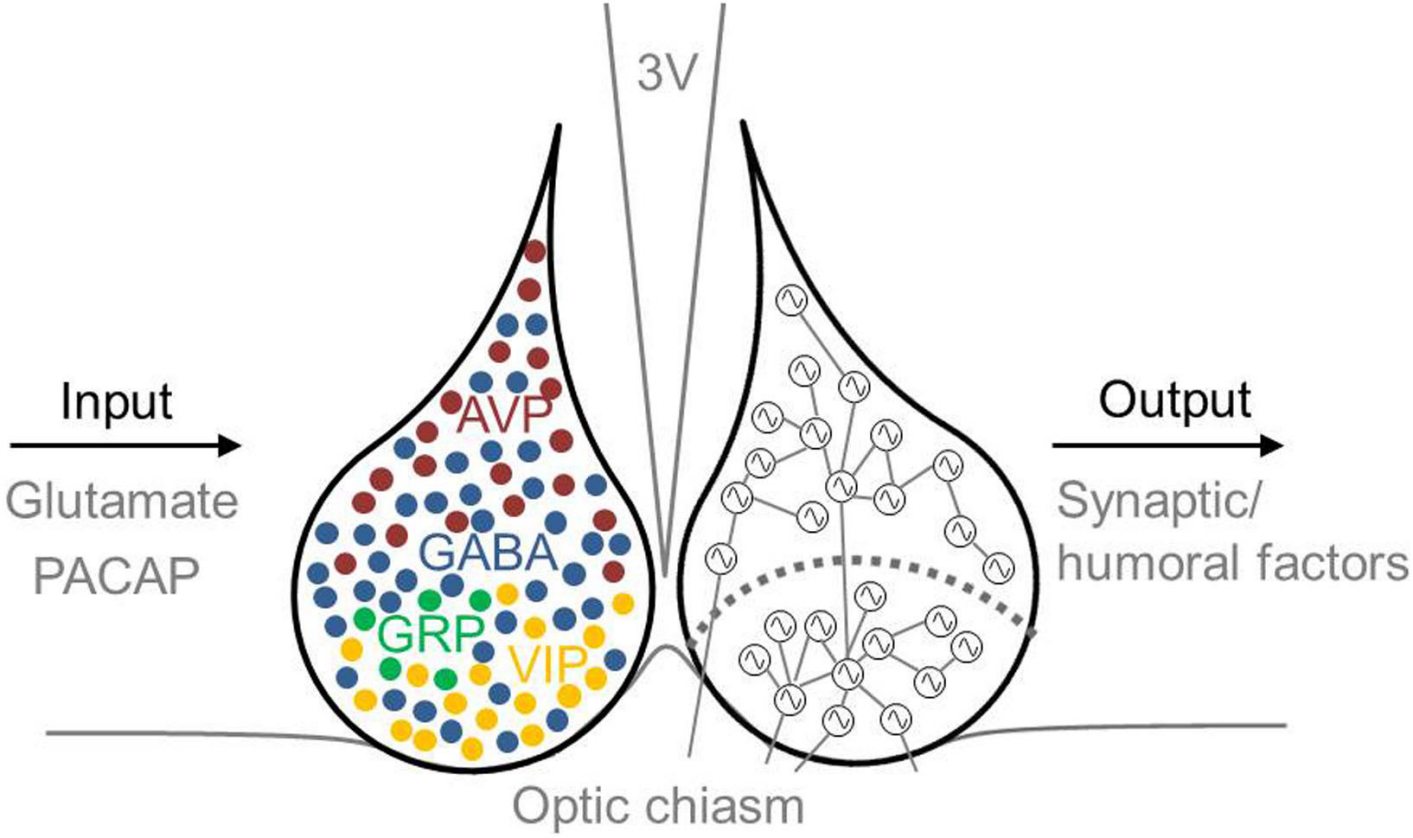
**Schematic illustration of the mammalian SCN, including inputs and outputs**. Input to the SCN is mediated by the neurotransmitters glutamate and PACAP, and output from the SCN is mediated by synaptic and humoral factors. The approximate locations of specific neurotransmitter-expressing neurons are indicated in the left nucleus. GABA (blue) is co-expressed in other neuronal cell types, including VIP- (yellow), AVP- (red), and GRP- (green) expressing neurons. AVP-expressing cells are located primarily in the dorsal SCN, and VIP-expressing cells are located primarily in the ventral SCN. The SCN depicted on the right schematically shows the ventral and dorsal SCN subdivision (the curved dotted line), and coupling between rhythmic SCN neurons is indicated by lines connecting the cells. Many neurons in the ventral SCN are directly innervated by afferent fibers arising from the optic chiasm, whereas far fewer neurons in the dorsal SCN receive direct light information input. The ventral SCN has direct input to the dorsal SCN, while the dorsal SCN is only sparsely innervated by the ventral aspect. Each neuron is depicted schematically as a cell-autonomous single-cell oscillator. 3V, third ventricle; AVP, vasopressin; GABA, γ-aminobutyric acid; GRP, gastrin-releasing peptide; PACAP, pituitary adenylate cyclase-activating polypeptide; SCN, suprachiasmatic nucleus; VIP, vasoactive intestinal peptide.

Several neurotransmitters play a role in the phase-synchronization of SCN neurons (Figure 2). For example, γ-aminobutyric acid (GABA) is the most prevalent neurotransmitter in the SCN. In the adult SCN, activation of GABA_A_ receptors causes an inhibitory response (84). This inhibitory effect of GABAergic signaling often plays a role in synchronizing neuronal networks within the brain (13, 85–87). Examining synchronization in SCN slices in the presence or absence of a GABA_A_ signaling blocker revealed that GABA plays a role in synchronizing SCN neurons. When slices prepared from a desynchronized SCN were treated with a GABA_A_ signaling blocker, the SCN neurons remained desynchronized; in contrast, in the absence of the blocker, the neurons became synchronized again (88). Although GABA acts predominantly as an inhibitory transmitter in the adult brain, it can play an excitatory role when coupled with the activity of the NKCC1 chloride pump (89, 90). GABAergic transmission is also excitatory in the dorsal SCN (47, 90, 91), and this excitation may play a role in communication between the ventral and dorsal SCN (47). Recently, we reported that GABAergic excitatory transmission is more prevalent in a desynchronized SCN than in a synchronized SCN (40 vs. 28%, respectively) (92), which suggests that the inhibitory/excitatory ratio of GABAergic activity plays a role in the phase-synchronization of individual SCN neurons.

The primary neurotransmitter in the ventral SCN is VIP. Intrinsically photoreceptive retinal ganglion cells (ipRGCs) contain the photopigment melanopsin and convey light information to the SCN via the RHT (93–95). VIP-containing neurons process light information received from the RHT and then transfer this information to the dorsal SCN (45, 96). The RHT contains both glutamate and pituitary adenylate cyclase-activating polypeptide (PACAP). The application of glutamate has an excitatory effect on SCN neurons, whereas glutamate receptor antagonists inhibit light-induced responses both *in vivo* and *in vitro* (97). Eliminating glutamate from ipRGCs impairs photo-entrainment of behavioral rhythm in mice (98); similarly, eliminating PACAP or its receptor also impairs photo-entrainment (99, 100). Although eliminating VIP reduces the light-induced upregulation of the clock gene *Per1*, photic induction of *Per1* is unimpaired in PACAP-deficient mice (99). Thus, VIP-expressing cells are critically important for relaying externally received light information to the SCN network. Eliminating VIP or the VIP receptor (VIP2R, also known as VPAC2) reduces SCN electrical activity (101), molecular rhythms (102, 103), and behavioral rhythms (104–107). In contrast, application of VIP mimics light-induced responses in the SCN (108, 109), drives long-lasting increased electrical activity in dorsal SCN neurons (110), and restores synchrony among SCN neurons in VIP-knockout mice (102–104). Finally, the SCN in VIP-knockout mice does not exhibit photoperiod adaptation. Taken together, these compelling findings indicate that VIP is important both for SCN neuronal synchronization and for the ability of the SCN to encode photoperiod-related information (111).

A subpopulation of neurons in the ventral SCN express GRP, and these neurons are important for conveying information regarding external light throughout the SCN (5, 8, 102, 103). In Syrian hamsters, *in vivo* microinjections of GRP into the third ventricle induces the expression of *c-fos*, *Per1*, and *Per2* in the dorsal SCN (8). GRP receptor-knockout mice have reduced light-induced phase shifts and reduced induction of *Per* and *c-fos*-expression in the dorsal SCN (112). The *in vitro* application of GRP to SCN slices induces a light-like phase shift in the SCN (113); moreover, applying GRP to SCN slices from VIP receptor-knockout mice increases synchrony among SCN neurons (5, 114).

The majority of neurons in the dorsal SCN express the neuropeptide vasopressin (AVP). This expression is rhythmic and is driven by the intrinsic molecular feedback loop in the core clock machinery. The *in vitro* application of AVP to SCN neurons isolated from VIP-deficient mice restores the rhythmicity and synchrony of the neurons (103). Furthermore, the expression pattern of AVP is different after entraining to a long photoperiod (i.e., in a desynchronized network) than after entraining to a short photoperiod (i.e., in a more synchronized network) (115, 116).

Connectivity within the SCN network is surprisingly plastic. In addition to seasonal plasticity, the SCN also exhibits a daily rhythm of synaptic connectivity. Measuring the firing rates of individual SCN neurons in the presence or absence of GABA_A_ receptor antagonists revealed that SCN connectivity is dependent upon GABAergic communication, and the strength of this connectivity can change in a matter of days or even hours (117). Recently, confocal microscopy studies revealed that synaptic changes occur in VIP-expressing neurons in the SCN’s retinorecipient region, but not in AVP-expressing neurons in the non-retinorecipient region (118). The authors hypothesized that remodeling of the synaptic connectivity in VIP-expressing neurons over a 24-h cycle might contribute to the ability of these neurons to adapt to light, thereby increasing the efficacy of photic transmission (118). In addition, during the subjective night, the neuron–glia network in the SCN undergoes morphological rearrangements (119), and during the day, axon terminal coverage of VIP-expressing neurons increases (119). These findings indicate that the SCN is a remarkably plastic structure that can efficiently adapt its network structure in response to changes in functional needs.

### Role of electrical coupling in synchronization of SCN neurons

The SCN network contains a large number of gap junctions that mediate electrical synchronization among SCN neurons (19, 120–123). Gap junction-mediated coupling improves the connectivity of neuronal networks (124, 125) and plays a role in the synchronization of several brain areas (121, 126–131). In the absence of chemical synaptic transmission, 24–26% of SCN neurons in rodents exhibit synchronous electrical activity (28, 121, 132); moreover, tracer-coupling experiments showed that 30% of SCN neurons are coupled via gap junctions (28, 120). In mice, gap junctions couple neurons in both the ventral and dorsal SCN, and clusters of coupled cells are restricted to each subdivision (120). In the mammalian CNS, the protein connexin-36 (Cx36) is a key component of gap junctions (131), and Cx36-knockout mice have significantly reduced electrical coupling in the SCN (121). Moreover, Cx36-knockout mice that were housed in continuous darkness had significantly reduced wheel-running activity compared to wild-type mice housed under the same conditions, suggesting that gap junctions play a role in the circadian organization of locomotor activity rhythms (121). Although immunofluorescence microscopy and immunogold labeling experiments confirmed that Cx36 is expressed abundantly in the postnatal SCN, tracer coupling and electrical coupling (i.e., coupled spiking) is relatively weak between SCN neurons in adult animals (122) compared to young animals (28, 120, 121). Interestingly, recent findings suggest that VIP increases gap junction-mediated coupling, as the application of VIP increased coupling efficiency between SCN neurons in which chemical coupling was blocked (123). This recent result supports the notion that the balance between chemical and electrical communication in the SCN is important for modulating synchronous rhythms, and disruption of only one form of communication can disrupt the circadian rhythmicity of the SCN and the periphery.

## SCN Neuronal Synchrony is Disrupted in Aging and Disease

### Aging and the SCN

**Figure 3:**
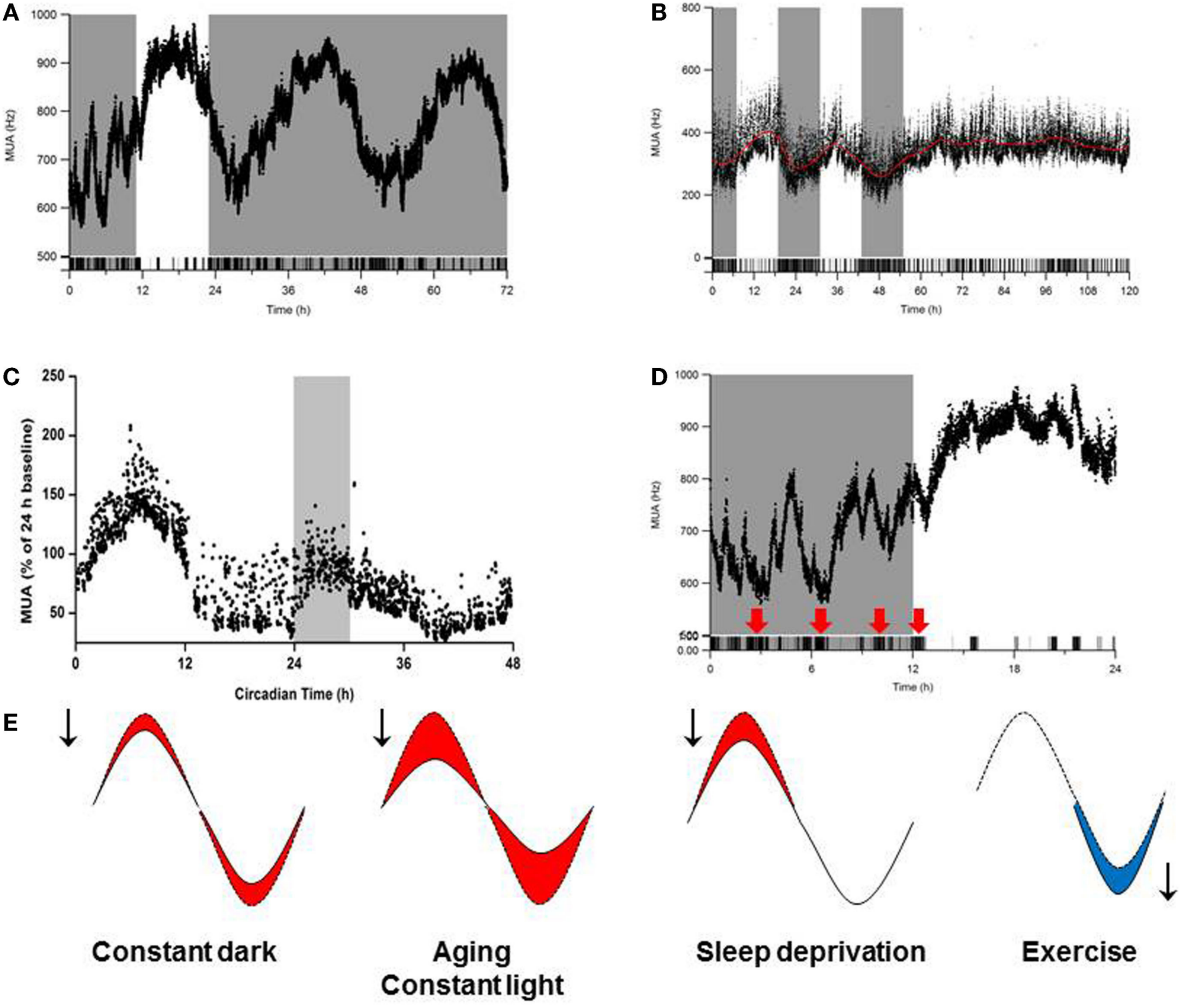
**Influence of environmental conditions on SCN amplitude**. Effects of **(A)** continuous darkness (212), **(B)** continuous light (212), **(C)** sleep deprivation (170), and **(D)** behavioral activity/physical exercise (254) on SCN neuronal activity measured using long-term *in vivo* recording of the SCN in freely moving mice. In **(A–D)**, the *x*-axis indicates (circadian) time (in hours), and the *y*-axis shows the electrical activity of the SCN ensemble (MUA, or multiunit activity) recording. **(E)** Schematic summary of the effects of continuous darkness, continuous light, sleep deprivation, and behavioral activity (exercise) on SCN neuronal activity. The red and blue areas under the curves represent a decrease or increase, respectively, in the SCN’s rhythm amplitude.

With aging, many species – including humans – experience changes in circadian timing. These changes are manifested as a reduction in the behavioral activity rhythm and in disruptions of the sleep–wake cycle (133–136). Given that the output of the SCN drives rhythms in behavior and physiology, these age-related disturbances in circadian rhythmicity could be caused by age-related deficits in the SCN. In support of this notion, transplanting fetal SCN tissue into the anterior hypothalamus of aged animals improves circadian rhythmicity in both hamsters (137, 138) and rats (139). *In vivo* recordings of electrical activity revealed reduced circadian amplitude in the SCN of middle-aged mice compared to young mice. Moreover, the amplitude of electrical activity rhythm in the subparaventricular zone – which receives input from the SCN – is substantially reduced in aged animals (140). Specifically, the amplitude of the SCN’s electrical activity rhythm in aged animals is approximately half of the amplitude in young animals (136) (Figure 3). This reduced amplitude cannot be explained simply by a loss of SCN neurons in aged mice (141) or rats (142), and experimental data suggest that age-related deficits in coupling between SCN neurons underlies the decrease in amplitude (136, 143).

Experiments have also revealed that SCN neurons in aged animals have an altered pattern of electrophysiological activity (136, 143–147). For example, the activity patterns of individual cells are less synchronized in the SCN of aged mice compared to young mice, and SCN neurons in aged mice even have anti-phasic activity (136). Computational studies found that decreased coupling in the aged SCN can lead to reduced synchrony among SCN neurons, thereby reducing the amplitude of the SCN’s network output (148). Physiologically, aging causes a clear change in the expression of neurotransmitters in the SCN. For example, the number of VIP- and AVP-expressing neurons is decreased in the SCN of aged rats (142, 149). In addition, the amplitudes of the circadian expression levels of *VIP* mRNA (150) and *VPAC2* mRNA (151) decline with aging. Functionally, *in vitro* patch-clamp recordings of SCN neurons from aged mice revealed that postsynaptic GABAergic currents are lower in both frequency (143) and amplitude (136) compared to recordings from young SCN neurons. Furthermore, the number of GABAergic synaptic terminals in the SCN of aged mice is reduced by 26% compared to young animals (152). These findings are relevant to humans as well, as neurotransmission decreases with aging. In elderly people, the number of VIP-expressing SCN neurons is reduced (153), and vasopressin levels are reduced in the SCN of elderly people from the age of 80 years (154). This age-associated decline in neurotransmission in the SCN could affect both the quality and degree of synchronization among SCN neurons.

Theoretically, a decline in the amplitude in the SCN’s rhythm can result from a decrease in synchronization and/or from a decrease in the amplitude of individual neurons (148). In mice, an age-related decrease in the SCN ensemble rhythms of *Per2* (155), *Clock*, and *Bmal1* expression (156–158) have been reported. SIRT1 is an NAD-dependent protein deacetylase that activates transcription of the *Clock* and *Bmal1* genes, thereby regulating circadian rhythms in tissues. In mice, an age-related decrease in SIRT1 levels in the brain corresponds with decreased levels of BMAL1 and PER2 and is associated with a longer intrinsic period, disrupted behavioral activity patterns, and impaired light entrainment (158). Several studies reported robust rhythms in *Per1* and *Per2* expression in the SCN of aged animals (159), and an increased amplitude of *Per2* expression has been reported in the SCN of aged mice (160).

### Sleep deprivation and the SCN

The sleep–wake cycle is regulated by both the circadian clock and a homeostatic mechanism that regulates sleep homeostasis (161). Homeostatic sleep pressure is reflected on an electroencephalogram (EEG) as slow-wave activity (SWA) during non-rapid eye movement (NREM) sleep. This homeostatic process can be manipulated by sleep deprivation; for example, sleep deprivation causes an increase in SWA in rats, hamsters, birds, and humans (162–168). During NREM sleep, SWA is negatively correlated with electrical activity in the SCN (169). Moreover, spontaneous transitions between NREM and REM sleep occur simultaneously with changes in SCN electrical activity (169), suggesting that sleep deprivation can directly affect the amplitude of the SCN’s electrical activity rhythm. This hypothesis was tested by performing simultaneous *in vivo* measurements of EEG and SCN firing rate in sleep-deprived rats. After 6 h of sleep deprivation, both SWA NREM sleep and REM sleep were significantly increased, and the SCN’s electrical activity was significantly decreased (170). These effects of sleep deprivation on SCN activity were long-lasting and could be measured at least 6 h after sleep deprivation (170) (Figure 3). In humans, disruptions in the quality and/or timing of sleep are common among the elderly and among individuals with neurodegenerative disorders (see below).

### Neurodegenerative disorders and the SCN

Patients with neurodegenerative disorders such as Alzheimer’s disease (AD), Huntington’s disease (HD), and Parkinson’s disease (PD) often exhibit perturbations in their 24-h activity patterns and in their sleep–wake cycles. Moreover, the expression patterns of clock genes are altered in the brains of patients with these neurodegenerative disorders compared to healthy subjects (171). In patients with AD, the loss of AVP is generally correlated with the severity of symptoms, suggesting a causal relationship (172). The 3xTg-AD mouse model of AD has disrupted circadian behavior and a decrease in VIP- and AVP-expressing neurons in the SCN (173, 174). Another model of AD, the APPxPS1 mouse, has disrupted sleep–wake rhythms compared to wild-type mice, but only a modest change in *Per2* expression in the SCN (175). R6/2 mice, a model of HD, develop progressively severe disruptions in behavior rhythms as the disease progresses (176), despite normal electrical output from the SCN (177). Two mouse models of HD and PD (the BACHD and ASO mouse lines, respectively) have disrupted circadian rhythms in locomotor activity, heart rate, and body temperature (178, 179). These two mouse lines also have reduced single-cell electrical activity in the SCN, although *Per2* expression is unaffected (178, 179).

Fragile X syndrome is the most common form of inherited mental retardation. Fragile X syndrome is caused by silencing of the *FMR1* gene and the subsequent loss of fragile X mental retardation protein (FMRP, also known as FXR1P) (180). Patients with fragile X syndrome suffer from sleep disorders (181, 182), and mice that lack either FMRP or FXR2P show a complete lack of behavioral rhythmicity (183). Interestingly, the SCN in Fmr1/Fxr2 double-knockout mice have normal expression rhythms of the *Per1*, *Per2*, *Bmal1*, and *Cry1* genes, and the SCN has high-amplitude electrical activity output (183). Molecular and electrophysiological data show that the SCN in the various mouse models of AD, HD, PD, and fragile X syndrome is functional, suggesting that the cause of the disease-associated perturbations in behavioral rhythmicity lie downstream of the SCN.

### Metabolic disorders and the SCN

Exposure to light during the subjective night alters the circadian rhythm of melatonin and cortisol levels (184, 185) and can alter sleep patterns (186, 187). Shift workers often develop severe circadian disruptions due to irregular timing of light exposure, which can increase the risk of developing type 2 diabetes or other conditions (188–191). Animal studies revealed that disrupting the circadian system by exposing animals to a shifted light–dark cycle or continuous light causes a range of symptoms that are similar to the effects of aging, including sleep disorders (192, 193), cardiovascular disorders (194–196), cognitive difficulties (197), and metabolic deficits (198–200). CLOCK-knockout mice have a dampened feeding rhythm and develop obesity and a host of other symptoms that are associated with metabolic disorder (199). The SCN plays a role in the regulation of energy homeostasis in both mice (201) and rats (202), and controls rhythms in energy metabolism by modulating rhythms in plasma glucose levels (203, 204), lipogenesis and lipolysis (205–207), and plasma leptin levels (208). Thermal ablation of the SCN in wild-type mice disrupts the circadian rhythmicity of energy intake, activity, and energy expenditure (209), as well as a loss of rhythm in plasma leptin levels (208). Lesioning the SCN induces mild obesity compared to sham-operated mice; however, hepatic insulin sensitivity decreases considerably, and basal glucose levels increase (209).

Repeated exposure to light during the subjective night causes increased body weight in mice (210, 211), and continuous exposure to light results in the complete loss of circadian rhythmicity in both energy metabolism and insulin sensitivity (212). Continuous exposure to light also reduces the amplitude of the SCN’s rhythm via desynchronization among the SCN neurons (213, 214). *In vivo* recordings of electrical activity in the SCN of freely moving mice that were continuously exposed to light revealed a 50% reduction in amplitude (212) (Figure 3). Remarkably, mice that are exposed continuously to light and fed a regular diet gain more weight than mice that are exposed to a standard light–dark cycle (12 h light:12 h dark) and fed a high-fat diet (212). A mixed-model analysis revealed that the reduction in the amplitude of the SCN’s output has a more severe effect on body weight than consuming a high-fat diet, thus underscoring the importance of robust SCN output for maintaining health. Disruptions in circadian rhythm and obesity are interrelated, and each disorder exacerbates the other, as a high-fat diet has a severely negative effect on the SCN’s ability to synchronize in response to light (215). Mice that genetically lack leptin (*ob/ob* mice) develop obesity and have altered phase-delaying capacity compared to heterozygous (*ob/*+) mice (216). Injecting *ob/ob* mice with leptin normalizes the photic response of the SCN, suggesting that leptin modifies light-induced responses in the SCN via an indirect pathway (216).

### Behavioral activity and the SCN

In addition to external light, several other stimuli provide input to the clock, and these inputs are known as non-photic stimuli. Examples include wheel-running activity (217), social interactions (218), dark pulses (219), and sleep deprivation (220). In general, non-photic stimuli induce behavioral activity, suggesting either that behavioral activity actually induces phase-shifting responses in the SCN (221) or that behavioral activity activates the same pathways. Interestingly, substances that are known to induce behavioral activity and/or influence neurotransmitter pathways involved in non-photic resetting – such as neuropeptide Y (NPY) (222), serotonin agonists (223), opioids (224), and short-acting benzodiazepines (225, 226) – induce a phase shift in the SCN’s rhythm.

The SCN receives its major inputs from two afferent pathways; the serotonergic tract provides input from the raphe nuclei, and the geniculohypothalamic tract provides input from the intergeniculate leaflet (IGL). Ablation of the serotonergic afferent SCN pathways attenuates the phase-shifting effects of several non-photic cues (227–230); moreover, increased levels of behavioral activity increase the levels of serotonin in both the SCN and the IGL (231, 232). In addition to behavioral activity, a phase shift can also be induced by arousal, and serotonin appears to play an important role in arousal-induced phase-shifting (230, 233). The geniculohypothalamic tract provides input to the SCN via the neurotransmitters NPY, GABA, enkephalin, and neurotensin (234–237). In rodents, wheel-running activity can induce the release of NPY in the SCN (238). The delivery of anti-NPY antibodies to the SCN attenuates the phase shift induced by novel wheel-running activity (239) and increases light-induced phase shifts (240). This finding suggests that NPY plays a role in both photic and non-photic resetting in the SCN (240).

Some non-photic stimuli suppress expression of *Per1* and/or *Per2* during the subjective day (241–245), and this suppression may underlie the SCN’s phase-shifting response to non-photic stimuli at the molecular level. Applying NPY (246–249) or the serotonin receptor agonist 8-OH-DPAT (250, 251) to SCN neurons *in vitro* decreases the neurons’ firing rate. At the *in vivo* level, recordings in the SCN of freely moving hamsters (252), rats (253), and mice (254) revealed that behavioral activity induces an immediate suppression of the SCN’s firing rate.

In mice, behaviorally induced suppression of SCN electrical activity is superimposed on the SCN’s circadian rhythm and occurs at all phases of the circadian cycle (254). The magnitude and duration of the suppression depend upon the intensity and duration of the behavioral activity, respectively. Even an ultra-short (i.e., briefer than 1 min) bout of behavioral activity can suppress the SCN’s firing rate to a rate similar to the rate achieved with longer bouts of activity. Less intense behaviors such as grooming, eating, drinking, and rearing can suppress electrical activity in the SCN by ~30%, and more intense activity such as locomotor activity can decrease electrical activity by up to 60% (254). The suppression in firing rate remains stable throughout the entire bout of behavioral activity, and switching between types of behavior (for example, from less intense activity to more intense activity) causes a change in the level of suppression. Because mice are nocturnal animals, their behavioral activity increases during the night; consequently, behaviorally induced suppressed firing decreases the trough of the SCN rhythm even further. On the other hand, during the day, mice are relatively inactive; consequently, the firing rate of the SCN is only barely suppressed during the peak of the rhythm. When a nocturnal animal is active during its resting phase, the peak in the SCN’s rhythm is lower, thereby resulting in a lower amplitude SCN rhythm. These studies suggest that scheduled behavioral activity can increase the SCN’s rhythmic amplitude, thereby improving peripheral rhythmicity. In support of this notion, voluntary exercise in aged mice increases the amplitude of the SCN’s firing rate *in vitro* and improves resynchronization of the SCN and peripheral systems to the light–dark cycle (255). Furthermore, mice lacking VIP or the VIP receptor show improved behavioral activity in response to scheduled locomotor activity (256, 257), and voluntary exercise improves circadian behavioral rhythmicity in a mouse model of HD (258). In humans, physical exercise accelerates the synchronization of sleep–wake rhythms to the external light–dark cycle (259) and improves health, mood, and performance (260–266).

## Comparison of the SCN between Nocturnal and Diurnal Animals

A long-standing – and inadequately addressed – question is whether the circadian clock is organized similarly or differently between nocturnal and diurnal animals. A comparison of the neuronal networks in the SCN of diurnal (*Acomys russatus*) and nocturnal (*Acomys cahirinus*) spiny mice revealed no differences with respect to neurotransmitter localization, SCN subdivisions, input fibers, or output fibers (267). Similar to nocturnal species, diurnal species have circadian rhythmicity in clock gene expression and electrical activity profiles in the SCN (268–278). On the other hand, diurnal and nocturnal species differ with respect to photic responses and photoperiod encoding. In the SCN of nocturnal species, ~25% of neurons are excited by light, whereas a smaller percentage of neurons are inhibited or silenced completely (29, 57, 279). In the SCN of diurnal species such as ground squirrels (280) and degus (281), the percentage of light-responsive cells is lower (~10%), and within the group of light-responsive cells, the proportion of light-suppressed cells is higher. Under LD 12:12, the overall rhythm in Fos-expression is similar between the nocturnal rat and the diurnal grass rat, however the spatial distribution of the Fos-expressing neurons differs. Whereas Fos-expression occurs in 40% of GRP containing SCN neurons in nocturnal rats (282), less than 1% of the GRP neurons in diurnal grass rats show Fos-expression (283). On the other hand, in grass rats, ~30% of AVP containing SCN neurons express Fos, while Fos-expression in AVP neurons in nocturnal rats is uncommon (284–286). Photoperiod studies suggest that light has a synchronizing effect on clock gene profiles and neuronal electrical activity in the SCN of mice, rats, and hamsters (65, 66, 287–291). However, the expression profiles of *Per1* and *Per2* in the SCN of diurnal grass rats do not adapt to short photoperiods (292). The SCN of diurnal and nocturnal species show a differential phase window for serotonin-mediated phase resetting (293). In nocturnal species, injections of serotonin receptor agonists 8-OH-DPAT or (+)8-OH-DPAT cause large phase advances of behavioral activity only during subjective midday (241, 294, 295) while in the diurnal *Arvicanthis*, injections of (+)8-OH-DPAT induce small phase advances during the subjective night (293). Importantly, in diurnal *Arvicanthis*, serotonin agonists strengthen, instead of oppose, the effects of light on the circadian system, which could be clinically relevant (293).

The SCN of nocturnal mice and diurnal grass rats respond differently to GABA. Activating GABA_A_ receptors during the subjective day causes a phase advance in the SCN of mice, whereas the SCN of grass rats exhibits a phase delay (296). In the SCN of mice, the rhythms in GRP and VIP are strengthened following continuous exposure to darkness. In contrast, in the SCN of diurnal grass rats, these rhythms do not become stronger; rather, they show a shift in their peak time of expression (297). Moreover, in the SCN of three-striped South Indian squirrels (*Funambulus palmarum*, a diurnal species), the phase relation of daily VIP and AVP rhythms differ from those found in nocturnal species (298). Recently, a study of the SCN in capuchin monkeys confirmed the presence of circadian oscillations in PER2 in the SCN of primates (299). Moreover, VIP-containing cells are present in the ventral SCN of capuchin monkeys (299), similar to VIP expression in nocturnal rodents (1, 3, 300, 301). In addition, AVP is present in both the ventral and dorsal SCN in capuchin monkeys (299), which is strikingly different than the expression patterns in the SCN of most nocturnal species studied to date (1, 3, 301–303).

Thus, the molecular clock is an evolutionarily preserved structure, and the SCN has both structural and functional similarities between nocturnal and diurnal species. On the other hand, the SCN of nocturnal and diurnal species differ with respect to their responsiveness to light, photoperiod, and neurotransmitters. These differences suggest fundamental differences between nocturnal and diurnal animals with respect to neuronal coupling in the SCN and synchronization mechanisms. These key differences should be considered carefully when making recommendations to improve the well-being of humans based largely on results obtained from studying nocturnal species.

## Summary

The functioning of the SCN depends on its intrinsic molecular machinery, as well as on its organization at the network level. Communication and synchronization among SCN neurons is essential for generating a robust rhythm at the SCN’s tissue level which is transmitted to other brain structures, thereby impacting many of our bodily functions. The SCN is influenced by environmental factors, the most important of which is light. In a short photoperiod, the SCN’s electrical activity rhythm is robust due to highly synchronized single-cell activity patterns, while in a long photoperiod, the SCN’s electrical output is dampened by reduced synchrony among individual cells. Photoperiod-induced changes in the expression of clock genes generally coincide with photoperiod-induced changes in the SCN’s electrical rhythm. Despite this similarity, spatial differences exist with respect to SCN clock gene expression, while the electrical activity rhythm has only small spatial differences among various regions within the SCN. With aging, the amplitudes of both single-cell rhythms and ensemble rhythms decline; however, reports of the amplitude of clock gene expression patterns in the aged SCN have been inconclusive. These points lead to the fundamental question of how the molecular feedback loop is correlated with the SCN’s electrical output.

The SCN clock is part of a larger brain network that includes areas involved in the sleep–wake cycle, energy metabolism, and behavioral activity. A dampened SCN rhythm is associated with reduced amplitude of the behavioral activity rhythm and can lead to metabolic disorders; vice versa, a disrupted behavioral rhythm (for example, induced by shift work or by food intake during the resting phase) can adversely affect the amplitude of the SCN’s rhythm. Increased levels of behavioral activity and voluntary exercise have been identified as potential strategies to boost the amplitude of the SCN’s rhythm, presumably by increasing cellular synchronization at the ensemble level. To be therapeutically effective for humans, it is important to investigate the effects of exercise on the SCN in diurnal species, particularly given that some differences have been found between nocturnal and diurnal animals. Whether – and how – the peripheral systems involved in behavioral activity and food intake influence the SCN in diurnal mammals remains largely unknown. Increasing our knowledge of the interplay between the SCN and the periphery in diurnal animals will increase our understanding of circadian-related disorders in humans and might lead to novel, effective recommendations for improving lifestyle patterns.

## Conflict of Interest Statement

The authors declare that the research was conducted in the absence of any commercial or financial relationships that could be construed as a potential conflict of interest.
